# Prognostic value of the combined preoperative plasma fibrinogen and systemic inflammatory indexes in ESCC patients

**DOI:** 10.1007/s12672-023-00763-7

**Published:** 2023-08-04

**Authors:** Honggang Cao, Hongtai Shi, Miaomiao Zhao, Zhenhua Liu, Jun Qian

**Affiliations:** 1 Department of Oncology, The Sixth Affiliated Hospital of Nantong University, Yancheng Third People’s Hospital, 75 Juchang Street, Yancheng, 224005 China; 2 Department of Radiation Oncology, The Sixth Affiliated Hospital of Nantong University, Yancheng Third People’s Hospital, 75 Juchang Street, Yancheng, 224005 China; 3 Department of Ultrasound, The Yancheng Clinical College of Xuzhou Medical University, The First People’s Hospital of Yancheng, 66 Renmin Road, Yancheng, 224005 China; 4 Department of Radiotherapy, The Yancheng Clinical College of Xuzhou Medical University, The First People’s Hospital of Yancheng, 66 Renmin Road, Yancheng, 224005 China; 5 Department of Thoracic Surgery, The Yancheng Clinical College of Xuzhou Medical University, The First People’s Hospital of Yancheng, 66 Renmin Road, Yancheng, 224005 China

**Keywords:** F-NLR, Fibrinogen, NLR, ESCC, Prognosis

## Abstract

The prognostic indexes based on the combination of preoperative fibrinogen and systemic inflammatory indexes may have greater predictive value in esophageal squamous cell carcinoma (ESCC). It was found that the predictive ability of F-NLR was more valuable than other systemic inflammatory indexes. The preoperative F-NLR score was closely related to the TNM stage, and could be used as an important independent prognostic index for patients with ESCC. Then the nomogram model constructed by F-NLR and TNM stage had higher prognostic ability than that of AJCC stage for ESCC patients. Preoperative F-NLR is a new independent prognostic index and a potential marker for treatment response monitoring in patients with ESCC.

## Introduction

In 2020, it is estimated that there are 604,100 new cases of esophageal carcinoma (EC) and 544,076 cases of deaths around the world, about half of whom are from Asia [1]. Esophageal squamous cell carcinoma (ESCC) is the main pathological type in China. Neoadjuvant chemoradiotherapy is the standard treatment for patients with locally advanced EC [2, 3], but many patients are still treated with surgical resection first. In recent years, despite advances in diagnosis and treatment of ESCC, long-term survival remains low after surgical resection [4]. Whether postoperative adjuvant therapy can bring survival benefits remains controversial. The AJCC TNM staging system has been widely applied in tumor risk stratification, survival outcome prediction, and development of diagnosis and treatment schemes. However, it still has certain limitations. Recently, many researches have found a large number of prognostic indexes related to EC patients, but most of the indexes are expensive in detection or in the experimental stage. Therefore, it is necessary to find reliable, simple and economic indexes.

Coagulation and systemic inflammatory response are important players in tumor development and metastasis [5, 6]. Fibrinogen is a kind of glycoprotein synthesized and secreted by hepatic cells, which is an important substance involved in the coagulation process. Recent studies have found that in lung carcinoma, EC, gastric carcinoma and colon carcinoma, the increase in plasma fibrinogen concentration can enhance the tumor invasiveness [7–11]. Therefore, hyperfibrinogenemia is correlated with the progression and shorter survival of patients with tumor [7–11]. In addition, many studies have also shown that systemic inflammatory indexes, such as neutrophil lymphocyte ratio (NLR), platelet lymphocyte ratio (PLR) and monocyte to lymphocyte ratio (MLR), can serve as effective indexes for predicting the prognosis of patients with malignancies [12–14]. Preoperative fibrinogen concentration or systemic inflammatory indexes are closely related to tumor progression, and they both have predictive value for the survival of patients with malignancies. There are certain limitations in displaying the tumor progression based on fibrinogen concentration or systemic inflammatory indexes alone. Therefore, prognostic indexes based on the combination of preoperative fibrinogen and systemic inflammatory indexes may have greater predictive value. In the present study, NLR, PLR and MLR were combined with preoperative fibrinogen, respectively, to synthesize new indexes (F-NLR, F-PLR and F-MLR). Then the predictive ability of preoperative F-NLR, F-PLR and F-MLR for the survival rate of ESCC patients was evaluated in two independent cohorts, so as to guide clinical practice and improve the clinical outcome of ESCC patients.

## Patients and methods

### Patients

ESCC patients undergoing radical resection in The First people’s Hospital of Yancheng from January 2010 to December 2015 were retrospectively analyzed. In addition, ESCC patients undergoing radical resection were enrolled from The Third People’s Hospital of Yancheng as a validation cohort. Inclusion criteria: (1) patients diagnosed with primary ESCC by postoperative pathology, (2) those undergoing radical resection, (3) those without receiving neoadjuvant radiotherapy or chemotherapy before surgery. In the primary cohort, a total of 674 patients were eligible for inclusion. In the validation cohort, a total of 215 patients were eligible for inclusion. Exclusion criteria: (1) those with R1 or R2 resection, (2) those with hematological diseases, autoimmune diseases or infections, (3) those with venous or arterial embolism or thrombosis within 3 months before surgery, and receiving continuous anticoagulation therapy, (4) those complicated with distant metastasis before surgery, (5) those with second primary tumor, (6) those who died during perioperative period. Based on the exclusion criteria, 186 patients were excluded in the primary cohort and 59 patients were excluded in the validation cohort. 29 patients lost to follow-up in the primary cohort, and 11 patients lost to follow-up in the validation cohort. Of the remaining 459 patients in the primary cohort and 145 patients in the validation cohort were included in our study. The staging system used for all patients is the 8th edition of the AJCC TNM staging system [15]. All patients signed the informed consent. All studies were conducted in accordance with the Declaration of Helsinki, and this retrospective study was approved by the Ethics Committee of The First people’s Hospital of Yancheng and Third People’s Hospital of Yancheng.

### Follow-up

After surgery, patients were reexamined every 3–6 months within 2 years, every 6 months in the next 3 years, and every 6–12 months thereafter. Follow-up content included routine laboratory examinations, including cervical/thoracic/abdominal enhanced CT, gastroscopy, cervical and abdominal lymph node ultrasound, upper gastrointestinal angiography, and PET/CT if necessary. When suspicious recurrence was found in the examination, biopsy of the lesion was conducted to confirm as far as possible, or chest X-ray and color Doppler ultrasound could be performed. Overall survival (OS) refers to the interval from the date of surgery to the date of death or the last follow-up.

### Definition of related indexes

At 1 week before the operation, peripheral venous blood was drawn from the patient for hematology analysis to detect the fibrinogen concentration, lymphocytes, neutrophils, platelets and monocytes. NLR is defined as the neutrophil count/lymphocyte count in peripheral venous blood. PLR is defined as the platelet count/lymphocyte count in peripheral venous blood. MLR is defined as the monocyte count/lymphocyte count in peripheral venous blood. According to receiver operating curve (ROC) analysis, the optimal cut-off value of NLR, PLR, MLR and fibrinogen was selected. It was found that the optimal cut-off value of NLR, PLR, MLR and fibrinogen was 1.95, 130, 0.25 and 3.50 g/L, respectively. F-NLR scoring criteria: Fibrinogen < 3.50 g/L and NLR < 1.95: 0 points of F-NLR; (fibrinogen ≥ 3.50 g/L and NLR < 1.95) or (Fibrinogen < 3.50 g/L and NLR ≥ 1.95): 1 point of F-NLR; fibrinogen ≥ 3.50 g/L and NLR ≥ 1.95: 2 points of F-NLR. F-PLR scoring criteria: fibrinogen < 3.50 g/L and PLR < 130: 0 points of F-PLR; (fibrinogen ≥ 3.50 g/L and PLR < 130) or (Fibrinogen < 3.50 g/L and PLR ≥ 130): 1 point of F-PLR; fibrinogen ≥ 3.50 g/L and PLR ≥ 130: 2 points of F-PLR. F-MLR scoring criteria: fibrinogen < 3.50 g/L and MLR < 0.25: 0 points of F-MLR; (fibrinogen ≥ 3.50 g/L and MLR < 0.25) or (Fibrinogen < 3.50 g/L and MLR ≥ 0.25): 1 point of F-MLR; fibrinogen ≥ 3.50 g/L and MLR ≥ 0.25: 2 points of F-MLR.

### Statistical methods

Chi-square test was used to analyze the correlation between F-NLR, F-PLR and F-MLR and clinicopathological characteristics and laboratory test indexes, respectively. Cox univariate analysis was applied to compare the results of the analysis between groups, so as to find the factors related to the prognosis of gastric cancer. If the candidate variables are a statistically significant difference in the univariate analysis, the factors will be further included in the cox multivariate analysis. More specific statistical methods are similar to our previous study [16]. SPSS 20.0 and R 3.6.2 were used for all statistical analyses.

## Results

### Construction of F-NLR, F-PLR and F-MLR based on the combination of preoperative fibrinogen and systemic inflammatory indexes

**Fig. 1:**
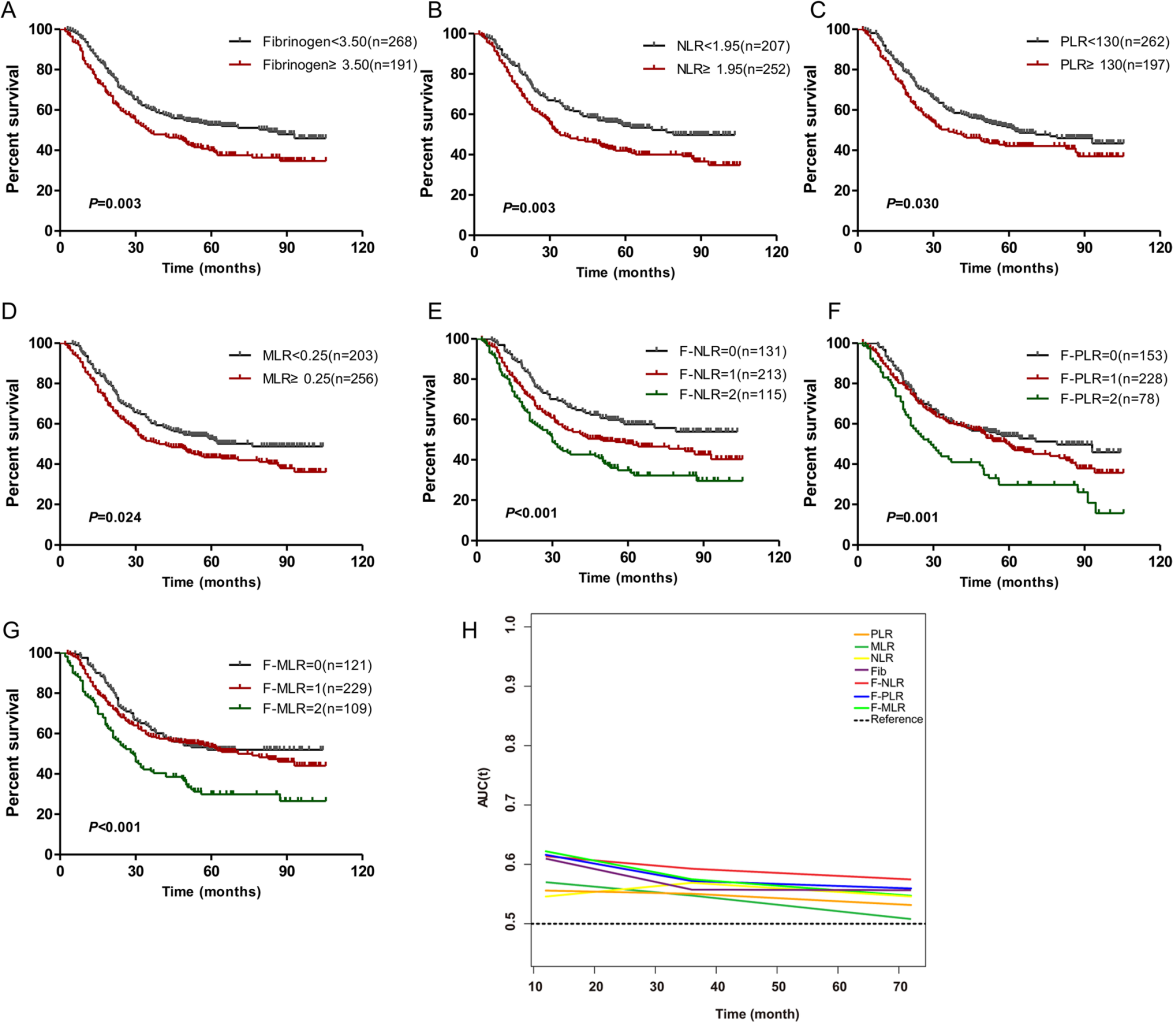
The prognostic significance of preoperative fibrinogen (**A**), NLR (**B**), PLR (**C**), MLR (**D**), F-NLR (**E**), F-PLR (**F**) and F-MLR (**G**) in ESCC in the primary cohort. **H** Predictive ability of the F-NLR in ESCC was compared with fibrinogen, NLR, PLR, MLR, F-PLR and F-MLR by time-depend ROC in the primary cohort

**Fig. 2:**
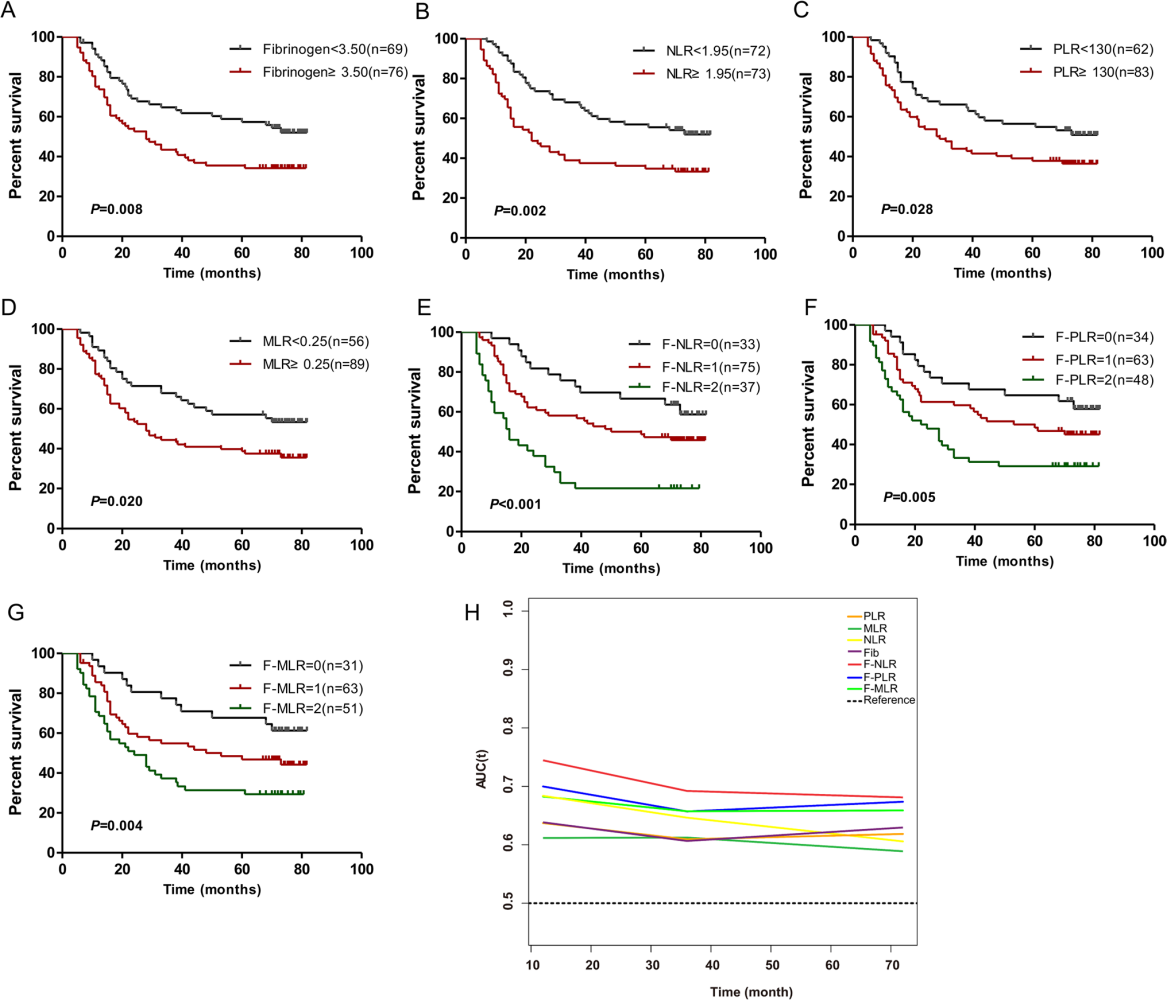
The prognostic significance of preoperative fibrinogen (**A**), NLR (**B**), PLR (**C**), MLR (**D**), F-NLR (**E**), F-PLR (**F**) and F-MLR (**G**) in ESCC in the validation cohort. **H** Predictive ability of the F-NLR in ESCC was compared with fibrinogen, NLR, PLR, MLR, F-PLR and F-MLR by time-depend ROC in the validation cohort

The baseline characteristics of primary cohort and validation cohort are shown in Table 1. Then the optimal cut-off value of fibrinogen, NLR, PLR and MLR were determined by the ROC curve of primary cohort. The indexes were divided into two groups (high-level and low-level) based on the cut-off value. In the primary cohort, Kaplan–Meier method was used to calculate the survival curve. It was found that ESCC patients with high fibrinogen, high NLR, high PLR or high MLR had significantly shorter postoperative survival time than those with low fibrinogen, low NLR, low PLR or low MLR (Fig. 1A–D). Then F-NLR, F-PLR and F-MLR were constructed as described above. The Kaplan–Meier method was used to calculate the survival curve in the primary cohort. It was found that patients with 2 points of F-NLR had a significantly shorter survival time than those with 0 and 1 point (Fig. 1E). There were similar results in F-PLR and F-MLR (Fig. 1F–G). Then by comparing the prognostic value of each index through the time dependent ROC analysis, it was found that the constructed F-NLR, F-PLR, and F-MLR had a larger area than a single systemic inflammatory index or fibrinogen, suggesting that the newly-constructed indexes have higher prognostic value (Fig. 1H, I). Among the newly-constructed indexes, the AUC of F-NLR was significantly larger than that of F-PLR and F-MLR, indicating that F-NLR may have greater prognostic value than other indexes. Then according to the analysis in the validation cohort, the results were basically the same as the primary cohort (Fig. 2A–I).

**Table 1:** Clinicopathological characteristics of patients with esophageal squamous cell carcinoma in primary cohort and validation cohort

Characteristic	Primary cohort (n = 459)		Validation cohort (n = 145)	
	No. of patients	%	No. of patients	%
Sex				
Male	350	76.3	112	77.2
Female	109	23.7	33	22.8
Age				
≤ 60	220	47.9	76	52.4
> 60	239	52.1	69	47.6
Tumor location				
Upper	31	6.8	14	9.7
Middle	296	64.5	91	62.8
Lower	132	28.8	40	27.6
Histological grade				
Well differentiated	25	5.4	9	6.2
Moderately differentiated	225	49.0	63	43.4
Poorly or not differentiated	209	45.5	73	50.3
T stage				
T1	125	27.2	29	20.0
T2	112	24.4	39	26.9
T3	215	46.8	75	51.7
T4a	7	1.5	2	1.4
N stage				
N0	263	57.3	74	46.1
N1	124	27.0	42	29.0
N2	55	12.0	21	14.5
N3	17	3.7	8	5.5
TNM stage (AJCC, 8th)				
I	108	23.5	22	15.2
II	195	42.5	68	46.9
III–IVa	156	34.0	55	37.9

### Prognostic value of preoperative F-NLR score in ESCC patients

It was found that the newly-constructed indexes F-NLR, F-PLR and F-MLR had significantly higher prognostic value than a single index for ESCC patients after surgery, and F-NLR had the highest prognostic value. Therefore, F-NLR was subjected to further analysis. The correlation analysis between F-NLR and clinicopathological characteristics was performed in the primary cohort, and it was found that F-NLR was only closely related to T stage and TNM stage, but not statistically related to sex, age, tumor location, histological grade and N stage (Table 2). In the primary cohort, a univariate analysis was conducted and it was found that gender, pathological grade, TNM stage, fibrinogen, NLR, PLR, MLR and F-NLR were all closely related to OS of ESCC patients. Then multivariate analysis also confirmed that TNM stage, fibrinogen, NLR, and F-NLR were independent prognostic factors for ESCC patients (Table 3). In the validation cohort, similar results were also obtained expect histological grade (Table 3). Histological grade was also an independent prognostic factor for ESCC patients in validation cohort.

**Table 2:** Baseline characteristics for patients with F-NLR score in primary and validation cohort

Clinical parameter	Primary cohort				Validation cohort			
	F-NLR = 0 (131)	F-NLR = 1 (213)	F-NLR = 2 (115)	*P*	F-NLR = 0 (33)	F-NLR = 1 (75)	F-NLR = 2 (37)	*P*
Sex				0.271				0.048^*^
Male	94	169	87		24	54	34	
Female	37	44	28		9	21	3	
Age				0.215				0.399
≤ 60	66	93	61		20	40	16	
> 60	65	120	54		13	35	21	
Tumor location				0.066				0.102
Upper	14	10	7		2	9	3	
Middle	72	145	79		25	48	18	
Lower	45	58	29		6	18	16	
Histological grade				0.071				0.135
Well	11	11	3		0	8	1	
Moderately	72	99	54		18	28	17	
Poorly	48	103	58		15	39	19	
T stage				0.003^*^				< 0.001^*^
T1	52	46	27		10	17	2	
T2	30	58	24		17	20	2	
T3	49	105	61		5	37	33	
T4a	0	4	3		1	1	0	
N stage				0.429				0.002^*^
N0	84	120	59		24	41	9	
N1	33	56	35		6	22	14	
N2	10	29	16		3	9	9	
N3	4	8	5		0	3	5	
TNM stage (AJCC, 8th)				0.004^*^				< 0.001^*^
I	44	41	23		9	12	1	
II	55	97	43		18	40	10	
III–IVa	32	75	49		6	23	26	
Fibrinogen				< 0.001^*^				< 0.001^*^
Fib < 3.5 g/L	131	137	0		33	36	0	
Fib ≥ 3.5 g/L	0	76	115		0	39	37	
NLR				< 0.001^*^				< 0.001^*^
NLR < 1.95	131	76	0		33	39	0	
NLR ≥ 1.95	0	137	115		0	36	37	
PLR				0.001^*^				< 0.001^*^
PLR < 130	92	113	57		22	34	6	
PLR ≥ 130	39	167	58		11	41	31	
MLR				< 0.001^*^				0.001^*^
MLR < 0.25	90	86	27		21	28	7	
MLR ≥ 0.25	41	127	88		12	47	30	

F-NLR: Fibrinogen-neutrophil lymphocyte ratio; NLR: neutrophil lymphocyte ratio; PLR: platelet lymphocyte ratio; MLR: monocyte to lymphocyte ratio

**Table 3:** Univariate and multivariate cox regression analyses for overall survival in patients with esophageal squamous cell carcinoma

Variables	Univariate analysis		Multivariate analysis	
	HR (95%CI)	*P* value	HR (95%CI)	*P* value
Primary cohort				
Sex: male vs. female	0.66 (0.48–0.90)	0.010^*^	0.79 (0.57–1.09)	0.156
Age: > 60 vs. ≤ 60	1.27 (0.99–1.63)	0.061		
Tumor location				
Lower vs. upper + middle	0.90 (0.78–1.04)	0.158		
Grade				
Poorly vs. Well + moderately	1.79 (1.39–2.30)	< 0.001^*^	1.68 (0.95–2.98)	0.074
T stage				
T2–4a vs. T1	3.01 (2.12–4.27)	< 0.001^*^	2.27 (1.58–3.28)	< 0.001^*^
N stage				
N1–3 vs. N0	2.80 (2.17–3.61)	< 0.001^*^	2.02 (1.54–2.65)	< 0.001^*^
TNM stage				
II–IVa vs. I	3.86 (2.57–5.80)	< 0.001^*^	3.57 (2.37–5.37)	< 0.001^*^
Fibrinogen: ≥ 3.5 vs. < 3.5	1.46 (1.14–1.88)	0.003^*^	1.35 (1.05–1.74)	0.019^*^
NLR: ≥ 1.95 vs. < 1.95	1.47 (1.13–1.89)	0.003^*^	1.33 (1.03–1.73)	0.031^*^
PLR: ≥ 130 vs. < 130	1.32 (1.03–1.69)	0.031^*^	1.19 (0.93–1.53)	0.164
MLR: ≥ 0.25 vs. < 0.25	1.34 (1.04–1.73)	0.025^*^	1.17 (0.90–1.51)	0.242
F-NLR				
1–2 vs. 0	1.62 (1.20–2.18)	0.002^*^	1.39 (1.03–1.88)	0.030^*^
Validation cohort				
Sex: male vs. female	0.66 (0.37–1.15)	0.139		
Age: > 60 vs. ≤ 60	0.93 (0.60–1.44)	0.742		
Tumor location				
Lower vs. upper + middle	1.02 (0.80–1.30)	0.877		
Grade				
Poorly vs. well + moderately	2.33 (1.48–3.68)	< 0.001^*^	1.87 (1.18–2.97)	0.008^*^
T stage				
T2–4a vs. T1	2.79 (1.39–5.58)	< 0.001^*^	2.16 (1.07–4.37)	0.032^*^
N stage				
N1–3 vs. N0	2.45 (1.57–3.83)	< 0.001^*^	1.84 (1.16–2.91)	0.010^*^
TNM stage				
II–IVa vs. I	3.24 (1.41–7.44)	0.006^*^	2.62 (1.13–6.10)	0.026^*^
Fibrinogen: ≥ 3.5 vs. < 3.5	1.80 (1.15–2.81)	0.010^*^	1.60 (1.02–2.51)	0.039^*^
NLR: ≥ 1.95 vs. < 1.95	1.96 (1.26–3.04)	0.003^*^	1.81 (1.14–2.87)	0.012^*^
PLR: ≥ 130 vs. < 130	1.64 (1.05–2.57)	0.031^*^	1.55 (0.98–2.44)	0.059
MLR: ≥ 0.25 vs. < 0.25	1.72 (1.08–2.74)	0.022^*^	1.50 (0.94–2.42)	0.092
F-NLR				
1–2 vs. 0	1.96 (1.10–3.48)	0.023^*^	1.84 (1.03–3.27)	0.039^*^

F-NLR: Fibrinogen-neutrophil lymphocyte ratio; NLR: neutrophil lymphocyte ratio; PLR: platelet lymphocyte ratio; MLR: monocyte to lymphocyte ratio

### Establishment and validation of nomogram prognostic model

**Fig. 3:**
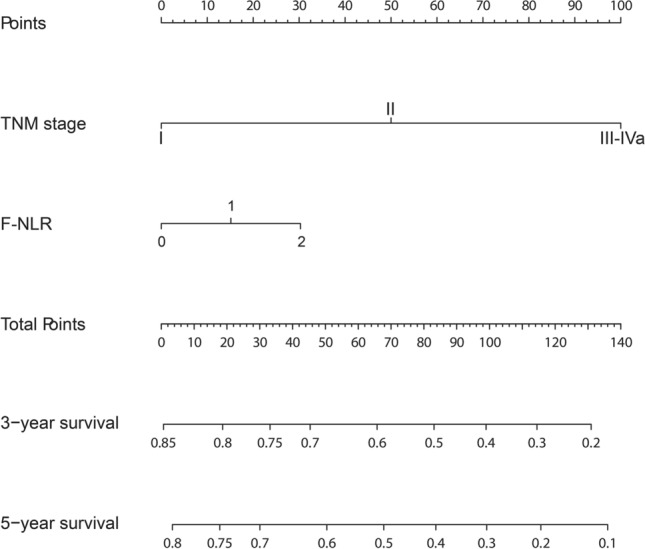
The nomogram integrating F-NLR and TNM stage for OS of patients with ESCC

**Fig. 4:**
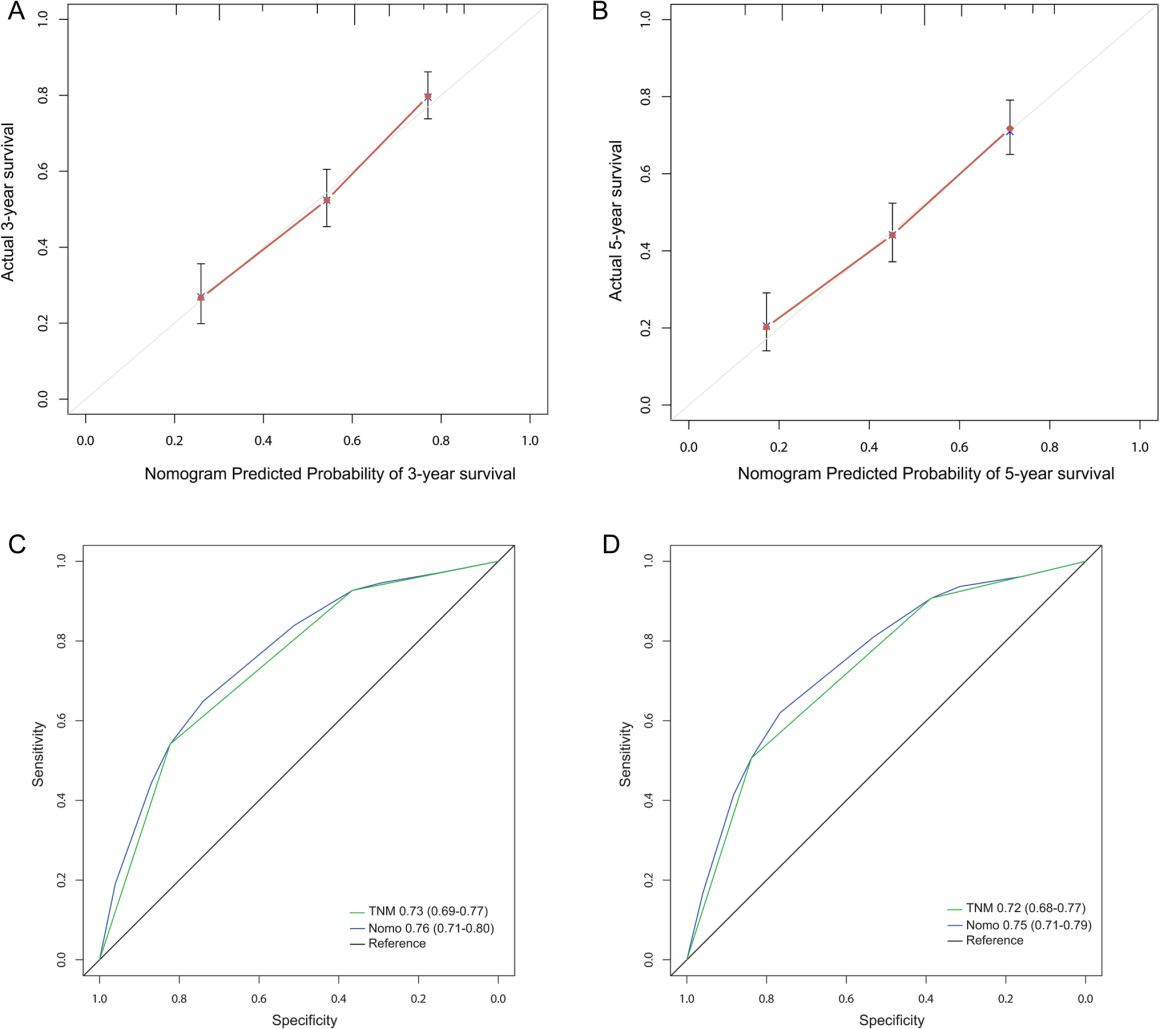
The 3-year (**A**) or 5-year (**B**) survival rate of ESCC patients predicted by nomogram is highly consistent with the actual observed values in the primary cohort. The ability of ROC analysis nomogram to predict the 3-year (**C**) or 5-year (**D**) survival rate of ESCC patients, the nomogram has a larger AUC than TNM staging in the primary cohort

**Fig. 5:**
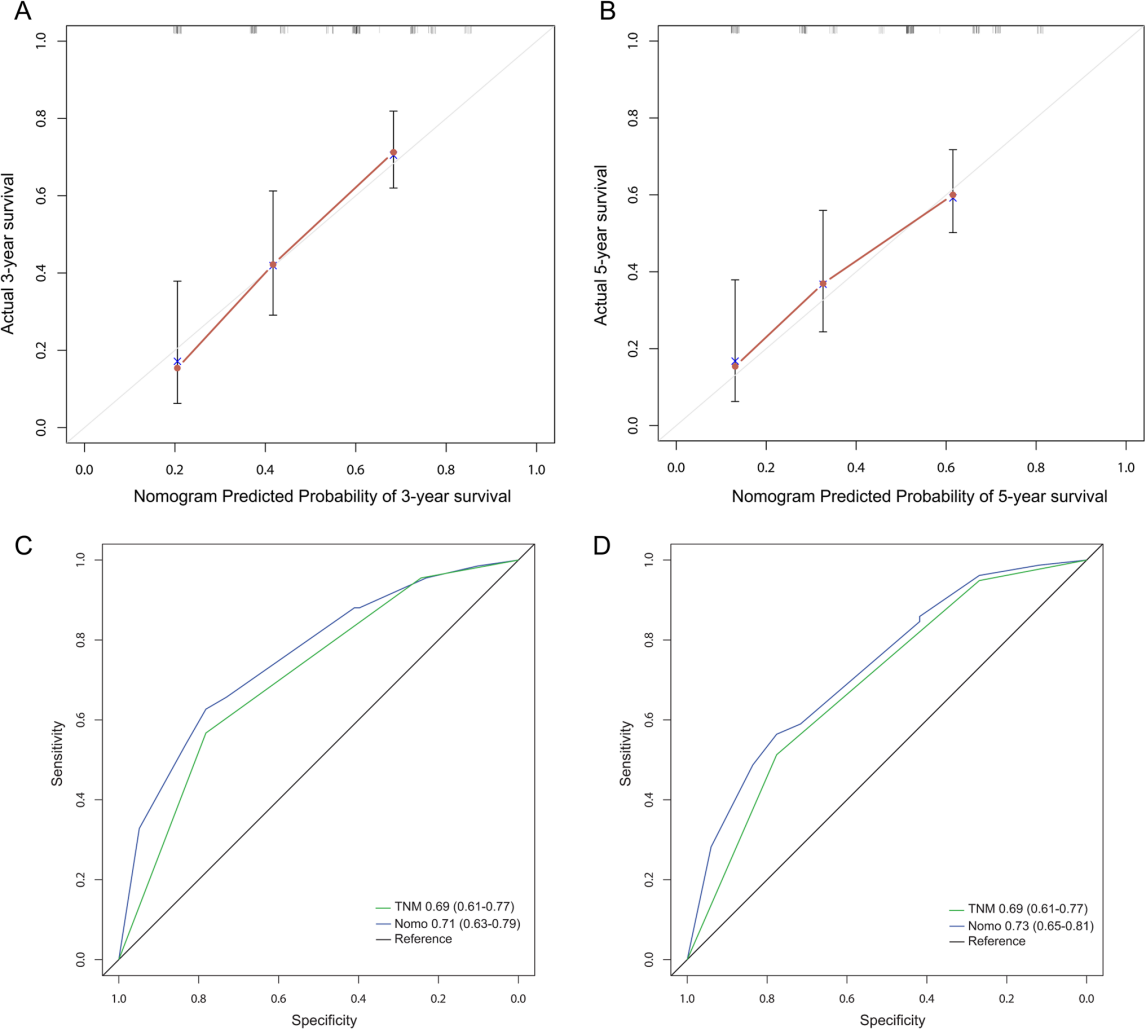
The 3-year (**A**) or 5-year (**B**) survival rate of ESCC patients predicted by nomogram is highly consistent with the actual observed values in the validation cohort. The ability of ROC analysis nomogram to predict the 3-year (**C**) or 5-year (**D**) survival rate of ESCC patients, the nomogram has a larger AUC than TNM staging in the validation cohort

F-NLR and TNM stage were used to establish the nomogram prognostic model for OS of patients with ESCC (Fig. 3), corresponding scores were assigned according to the weight of each factor, and the corresponding 3- and 5-year survival rate was predicted based on the total score. The calibration chart showed that the 3- and 5-year OS predicted by the model was highly consistent with the actual rate (Fig. 4A, B). According to the ROC curve analysis, the AUC of the nomogram model was significantly larger than that in the eighth edition of AJCC staging (Fig. 4C, D), indicating that the nomogram model is more accurate in evaluating the OS of ESCC patients. The nomogram prognostic model was then validated in another independent validation cohort. The calibration chart also showed that the predicted survival rate was consistent with the actual observed (Fig. 5A, B). In addition, the AUC of the nomogram model was also significantly larger than that of TNM stage (Fig. 5C, D).

## Discussion

Despite great progress in the diagnosis and treatment of EC, the prognosis of ESCC patients remains unsatisfactory. Accurate preoperative prognosis judgment and treatment plan play an important role in the individualized treatment of patients. Tumor progression and prognosis are not only determined by the characteristics of the tumor itself (such as differentiation degree, size and invasiveness), and some related factors of the patient itself (such as immune response) are also important. In recent years, many researches have shown that systemic inflammatory indexes and fibrinogen in plasma are related to the progression and prognosis of various malignancies, which can be used as effective indexes to judge the prognosis. Previous studies on fibrinogen and systemic inflammatory indexes in tumor prognosis separated the two aspects. In this study, preoperative fibrinogen and systemic inflammatory indexes were combined to construct F-NLR, F-PLR and F-MLR. It was found that F-NLR was more valuable in evaluating prognosis. Subsequently, the relationship between F-NLR and the clinicopathological characteristics and prognosis of ESCC patients was discussed. The results showed that the preoperative F-NLR score was closely related to the TNM stage, and could be used as an important independent prognostic index for patients with ESCC. Then the nomogram model constructed by F-NLR and TNM stage had higher prognostic ability than that of AJCC stage for ESCC patients.

Fibrinogen is an acute phase reactive protein synthesized by the liver. As a coagulation factor in plasma, it participates in the coagulation process, which plays an important role in platelet aggregation, plasma viscosity increase, vasoconstriction, and growth factor secretion [17]. In clinic, when malignancies or inflammation occur, the level of fibrinogen in plasma often increases, and fibrinogen can be converted into fibrin under the action of activated thrombin. Relevant studies have shown that fibrinogen plays a vital role in tumor occurrence, promotion of angiogenesis, and hematogenous metastasis of tumor cells [18, 19]. Tumor cells themselves can also synthesize fibrinogen, which can act as a regulator of tumor cell proliferation and metastasis [20, 21]. Yamaguchi et al. found that in lung carcinoma patients, tumor cells can synthesize interleukin-6, which can promote the secretion of fibrinogen, affecting the development of tumors [22]. By interacting with FGF2 and VEGF, fibrinogen can regulate the adhesion, proliferation and migration of tumor cells [23]. Related studies have also manifested that high concentrations of fibrinogen can induce epithelial and mesenchymal transformation, which can confer the metastasis, infiltration and multi-drug resistance on tumor cells [24, 25]. Many studies have found that the level of fibrinogen in plasma is related to the progression and prognosis of ESCC [26–28].

Recent studies have found that systemic inflammation also is important in tumor progression and prognosis [29]. Some researches showed that inflammation-related cells can secrete cytokines into the tumor microenvironment [30, 31]. Moreover, some systemic symptoms related to carcinoma, such as weight loss, cachexia and anemia, are also affected by systemic inflammation [32]. Therefore, in terms of prognostic factors, indexes related to inflammation have received extensive attention. As important immune cells in the inflammatory response, lymphocytes and neutrophils play an important role in tumor progression. In the anti-tumor immunity, lymphocytes play an important role in immune surveillance and immunoediting, which can inhibit the proliferation and migration of tumor cells [6]. T lymphocytes have the biological function of killing target cells and can induce tumor cell apoptosis [33]. Neutrophils are the most abundant white blood cells in the blood circulation, which play a highly important role in the body's non-specific cellular immune system. High-level circulating neutrophils are related to chemokines, growth factors and proteases, which are essential for angiogenesis [34]. In terms of reflecting the influence of inflammation or immune response on tumor progression, the evaluation value of lymphocyte or neutrophil count alone has its own limitations. Lymphocyte count is a favorable prognostic factor, while neutrophil count is an unfavorable prognostic factor. The variable NLR, the combination of neutrophil and lymphocyte count, can expand their respective effects and expand the prognostic value for patients with malignancies. Therefore, many studies have shown that in a variety of solid tumors including ESCC, NLR can be used as an effective index for prognosis [12–14].

According to a large number of basic and clinical studies above, fibrinogen and NLR are closely related to tumor progression and prognosis. In recent years, some studies have shown that the scoring system composed of fibrinogen and NLR is also of high value in predicting the survival of patients with malignancies [35–40]. Previously, three studies evaluated the prognostic value of F-NLR in ESCC, and all confirmed that F-NLR has a good prognostic judgment for ESCC [37, 39, 40]. However, these three studies only evaluated F-NLR, and did not evaluate the prognostic value of F-PLR and F-MLR. Moreover, these three studies were independent single-center studies, and this study was a two-center study, so the conclusions were more reliable. In addition, a nomogram model was constructed in this study based on F-NLR and TNM stage to further improve the prognostic prediction of ESCC patients.

## Conclusion

Preoperative F-NLR is a new independent prognostic index in patients with ESCC. The nomogram model based on the F-NLR and TNM stage can predict the survival of patients with ESCC more objectively and reliably than the traditional TNM staging system, which helps clinicians shunt ESCC patients according to the risk of death, and develop more accurate and prompter individualized therapeutic regimens as early as possible.

## Data Availability

The original datas presented in the study are included in the article/supplementary material, further inquiries can be directed to the corresponding author.
